# Performance of HPV testing and liquid-based cytology after negative colposcopy in women with ASC-H, HSIL or AGC cytology

**DOI:** 10.61622/rbgo/2026rbgo62

**Published:** 2026-09-23

**Authors:** Cirbia Silva Campos, Adriane Cristina Bovo, Bruno Fonseca, Iara Vidigal Santana, Marcio Antoniazzi, Sandra Maniçoba Palopoli, Julio Cesar Possati-Resende, Luiz Carlos Zeferino

**Affiliations:** 1 Universidade de Campinas Faculdade de Ciências Médicas Campinas SP Brazil Faculdade de Ciências Médicas, Universidade de Campinas, Campinas, SP, Brazil.; 2 Hospital de Câncer de Barretos Departamento de Prevenção de Câncer Campo Grande MS Brazil Departamento de Prevenção de Câncer, Hospital de Câncer de Barretos, Campo Grande, MS, Brazil.; 3 Departamento de Prevenção de Câncer Barretos SP Brazil Departamento de Prevenção de Câncer, Barretos, SP, Brazil.; 4 Departamento de Patologia Barretos SP Brazil Departamento de Patologia, Barretos, SP, Brazil.; 5 Departamento de Prevenção de Câncer Nova Andradina MS Brazil Departamento de Prevenção de Câncer, Nova Andradina, MS, Brazil.

**Keywords:** Human papillomavirus DNA tests, Human papillomavirus 16, Papillomavirus infections, Cytology, Cervical neoplasms, Mass screening, Colposcopy, Uterine cervical dysplasia

## Abstract

**Objective:**

To evaluate the performance of HPV testing and liquid-based cytology (LBC) in detecting HSIL+ (CIN2, CIN3, AIS, or cancer) after negative colposcopy in women screened with ASC-H, HSIL or AGC cytology.

**Methods:**

We conducted a multicenter clinical trial (NCT05120167) involving 600 women referred for colposcopy due to ASC-H or worse cytology between October-2021 and October-2022. After excluding positive colposcopy cases, 257 women with negative colposcopy underwent HPV DNA testing and LBC. Histopathological diagnosis after excision of the transformation zone or 18-month negative follow-up served as the reference standard. Sensitivity, specificity, predictive values (PV), and accuracy were calculated for each test individually and combination.

**Results:**

A total of 227 women completed the study. HSIL+ was diagnosed in 15.4% (35/227), including two superficially invasive carcinomas. HPV testing alone demonstrated the highest sensitivity (94.3%) and negative PV (98.6%), but lower specificity (73.4%) and positive PV (39.3%). When restricted to HPV16 and/or 18, specificity increased to 94.8% and PPV to 57%. LBC showed balanced performance, with sensitivity of 80%, specificity of 84.9%, and PPV of 49.1%. Combining HPV and LBC using an "OR" rule yielded the highest sensitivity (97.1%) and NPV (99.2%), whereas the "AND" rule provided the highest specificity (91.7%), PPV (62.8%), and overall accuracy (89.4%).

**Conclusion:**

A substantial proportion of HSIL+ lesions persist despite negative colposcopy. HPV testing effectively rules out disease, and combining with LBC enhances diagnostic safety and accuracy. This dual-test approach offers a practical strategy to guide management and reduce diagnostic failures in women with ASC-H or worse cytology.

## Introduction

Cervical cancer remains a major cause of global mortality and is associated with persistent infection by oncogenic high-risk human papillomavirus (hr-HPV).^(1)^ Control strategies include HPV vaccination, periodic screening for precursor lesions, and timely access to appropriate treatment.^(2)^ Conventional cytology remains the most widely used method for screening method globally. Women with abnormal results or at increased risk of cervical lesions are referred for colposcopy,^(3,4)^ an essential tool for identify suspicious areas and guiding biopsies.

Most subclinical cervical lesions arise in the transformation zone (TZ), near the squamocolumnar junction (SCJ) and the external cervical os. In some cases, however, the SCJ is not fully visible, which complicates the exclusion of lesions located in the endocervical canal.^(3,5)^

Current guidelines recommend clinical management based on the combined results of screening tests and colposcopy.^(3,4,6,7)^ However, discrepancies between these assessments do occur, creating diagnostic challenges. A negative colposcopy following screening cytology showing significant abnormalities, such as "atypical squamous cells, cannot exclude high-grade squamous intraepithelial lesion" (ASC-H), "high-grade squamous intraepithelial lesion" (HSIL), or "atypical glandular cells" (AGC), is of particular concern. This scenario raises the possibility of an occult endocervical lesion that requires further investigation.^(3,6,7)^

Incorporating complementary diagnostic methods has therefore gained increasing importance. HPV DNA testing offers high sensitivity and excellent negative predictive value (NPV) for HSIL+ (CIN2, CIN3, AIS, or cancer),^(9)^ whereas liquid-based cytology (LBC) provides more balanced diagnostic performance and higher specificity.^(10)^ Using these tests in combination with negative colposcopy may increase diagnostic safety and optimize clinical decision-making. Evaluating the role of these integrated strategies to detect occult endocervical lesions is thus essential to reduce diagnostic failures and support future recommendations.

## Methods

This was a multicenter, controlled clinical trial (NCT05120167; https://clinicaltrials.gov/study/NCT05120167) designed to evaluate the performance of additional tests following negative colposcopy in women with screening cytology showing significant atypia (ASC-H or worse: ASC-H, HSIL, or AGC) for the detection of HSIL+.

The sample size calculation assumed an 80% confirmation rate of diagnoses by the evaluated test, with 95% power, a 5% significance level, and up to 20% losses. A total of 250 women with negative colposcopy was therefore required. This number was reached after including 600 women initially referred for colposcopy due to ASC-H or worse cytology performed within the preceding 12 months. Recruitment occurred between October 2021 and October 2022 at four reference centers of Barretos Cancer Hospital (HCB), located in Barretos (SP), Campinas (SP), Campo Grande (MS), and Nova Andradina (MS), Brazil. Enrollment was stratified by age group (25-34, 35-49, and ≥ 50 years) and screening cytology category (ASC-H, HSIL or AGC) (Figure 1).

**Figure 1:**
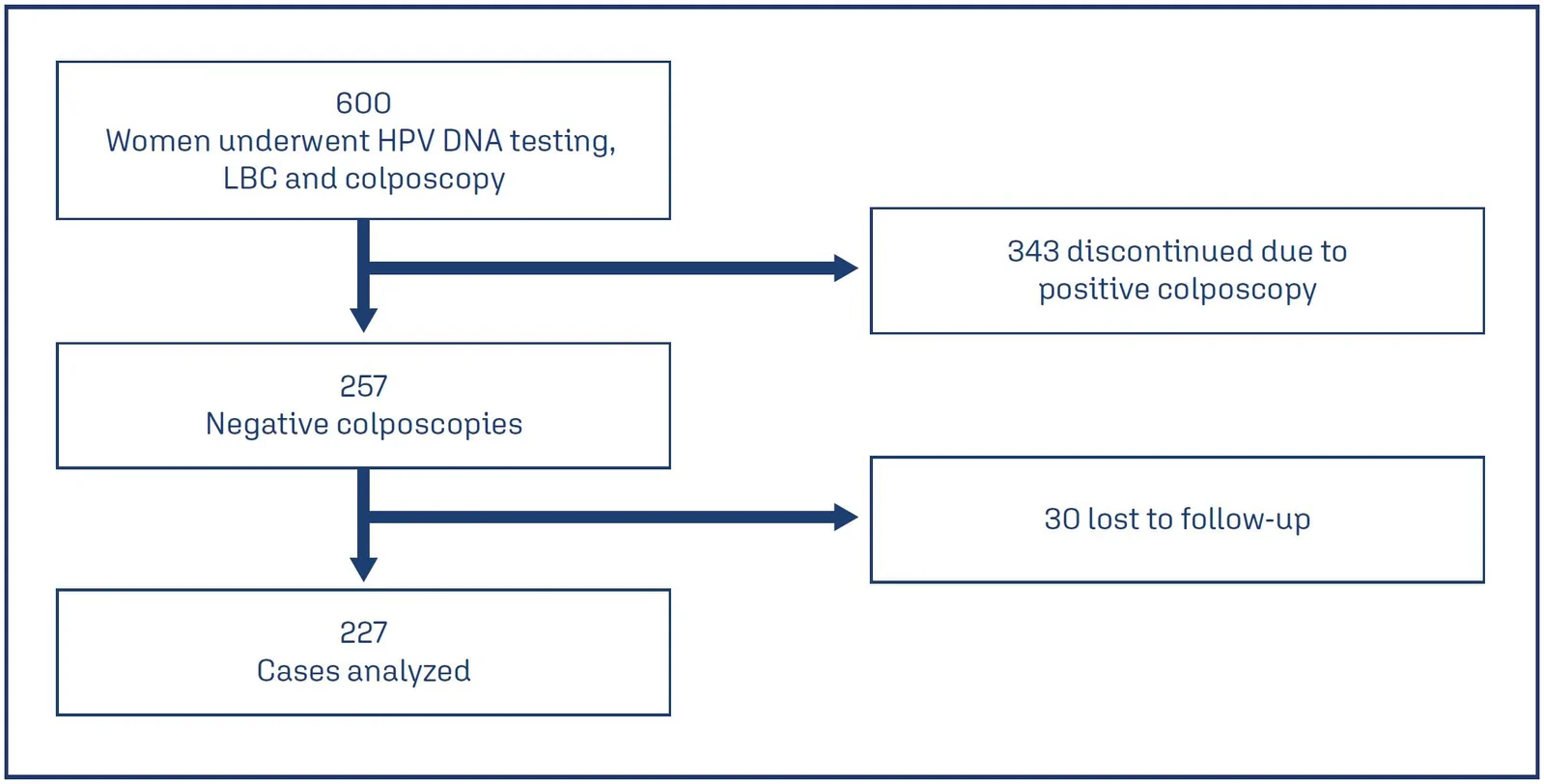
Case selection flowchart: 600 women with screening cytology showing ASC-H, HSIL, or AGC underwent colposcopy. Participants with positive colposcopy findings were discontinued (n=343), and 257 with negative colposcopy remained. There were 30 losses in the follow-up (11,7%) resulting in 227 cases analyzed for final diagnosis

All participants provided written informed consent. Cervical samples were collected using a standard brush (Rovers^®^ Cervex-Brush^®^ Combi) for HPV testing and liquid-based cytology (LBC), followed by colposcopy. Women with positive colposcopy findings (n=343) were discontinued and managed according to routine clinical protocols. The study proceeded with 257 women who had negative colposcopy results, including a negative vaginoscopy (Figure 1).

The HPV test used (COBAS HPV test, Roche Diagnostics, Pleasanton, CA, USA) detects 14 hr-HPV types, with individual genotyping for HPV16 and HPV18 and pooled detection for the remaining 12 other types (12OT).^(11)^ LBC was performed using the BD SurePath methodology (Becton, Dickinson and Company, Franklin Lakes, NJ, USA).^(12)^ Cytological diagnoses followed the Bethesda System 2014^(13)^ and the Brazilian nomenclature for cytopathology reports.^(14)^ Colposcopy findings were described according to the International Federation for Cervical Pathology and Colposcopy (IFCPC) 2011 classification.^(8)^ Histopathological evaluation was performed according to routine institutional protocols by experienced pathologists responsible for pathological analysis from all study centers. Histological diagnoses followed the Lower Anogenital Squamous Terminology (LAST) criteria and were classified as negative, LSIL (CIN1), HSIL (CIN2, CIN3), AIS, or cancer.^(15)^

Performance analyses of the tests, individually or in combination, used histopathological diagnosis from excision of the transformation zone (EZT) as the reference standard. For cases without EZT indication, a negative diagnosis was defined after two follow-up visits within 18 months (colposcopy and LBC without abnormalities) or one negative follow-up visit plus a negative HPV test after 12 months. Results of HPV testing and LBC were analyzed according to age groups, screening cytology category, and detection of HSIL+. Sensitivity, specificity, positive predictive value (PPV), NPV, and accuracy were calculated. Unsatisfactory or invalid tests were classified as false positives (FP) or false negatives (FN).

Statistical analysis was performed using StatsDirect 3.0 software (Wirral, UK, www.statsdirect.com), with *p* < 0.05 considered statistically significant. The study was approved by the Ethics Committee of HCB, PIO XII Foundation (approval no. 2183/2021, issued August 13, 2021; CAAE:49006721.5.0000.5437).

## Results

The mean interval between the screening cytology and the study procedures was 103 days, with 80% of cases occurring within 120 days and 95% within 180 days. Women that completed all study procedures were 227 and the loss of follow-up rate was 11.7% (Figure 1). The mean age of participants was 48 years, with 49% aged 50 years or more, and 75% had ASC-H as their screening cytology. HPV-positive results were more frequent in the 25-34 age group compared with older women (50% vs. 34-37%), *p* = 0.044), and among those with ASC-H screening cytology compared with HSIL or AGC (34% vs. 22%, p = 0.015), than positive LBC results (Table 1).

**Table 1:** Distribution of 227 HPV and liquid-based cytology (LBC) results following negative colposcopies, stratified by age group and screening cytology

Variable	n(%)	Post-negative colposcopy test results				p-value
		HPV test		LBC		
		Positive (n=84)	Negative (n=143)	Positive (n=57)	Negative (n=170)	
		n(%)	n(%)	n(%)	n(%)	
Age group						
25-34 years	26(11)	13(50)*	13(50)	6(23)	20(77)	0.044
35-49 years	90(40)	33(37)*	57(63)	23(26)**	67(74)	0.107
>50 years	111(49)	38(34)	73(66)	28(25)	83(75)	0.142
Screening cytology						
ASC-H	170(75)	57(34)*	113(66)	37(22)**	133(78)	0.015
HSIL	34(15)	20(59)	14(41)	17(50)	17(50)	0.465
AGC	23(10)	7(30)*	16(70)	3(13)	20(87)	0.142

LBC, liquid-based cytology; ASC-H, atypical squamous cells, cannot exclude HSIL; HSIL, high-grade squamous intraepithelial lesion; AGC, atypical glandular cells. HPV test detected 14 hr-HPV types, with genotyping for HPV16 and HPV18 and pooled detection for 12 other types. LBC positive diagnosis included ASC-H or worse. Statistics: Chi-square or Fisher exact test

The final HSIL+ detection rate among women with negative colposcopy was 15.4% (35/227), including two superficially invasive carcinomas (Stage IA1: one squamous and one glandular). An additional 14 cases of LSIL (CIN1) were also identified. As shown in table 2, 40.2% (33/82) of HPV-positive tests were associated with HSIL+, compared with only 1.4% (2/143) when HPV testing was negative (*p* < 0.0001). HPV16 and/or18 were detected in 23 cases, 13 of which (56.5%) had HSIL+, whereas detection of the other 12 high-risk HPV types (12OT) was associated with HSIL+ in 33.9% (20/59) of cases (*p* = 0.061). LBC results classified as ASC-H or worse were associated with HSIL+ in 50.9% (28/55), compared with only 4.1% (7/170) when LBC results were negative, ASC-US, or LSIL (*p* < 0.0001). Among these seven false-negative LBC cases, four were diagnosed as CIN3 and one as superficially invasive carcinoma. The remaining two cases were initially classified as ASC-US but were later confirmed as CIN2. For LBC ASC-H category, 32.4% (11/34) correlated with HSIL+ diagnoses.

**Table 2:** Association between HPV DNA and LBC results after negative colposcopy and the detection of HSIL+ (CIN2, CIN3, AIS or cancer) in 225* women

Test result*	HSIL+ detection		Total
	No(n=190)	Yes(n=35)	(n=225)
	n(%)	n(%)	n
HPV negative	141(98.6)	2(1.4)	143
HPV positive	49(59.8)	33(40.2)	82
HPV16 and/or 18	10(43.5)	13(56.5)	23
12 Other HPV types	39(66.1)	20(33.9)	59
LBC negative, ASC-US or LSIL	163(95.9)	7(4.1)	170
LBC positive	27(49.1)	28(50.9)	55
ASC-H	23(67.6)	11(32.4)	34
HSIL	4(21.1)	15(78.9)	19
AGC or AIS	0(0)	2(100)	2

* One case for each test yielded invalid or unsatisfactory sample (2/227, 0.9%). Positive results for both HPV DNA testing and LBC were significantly associated with HSIL+ detection (p<0.0001). HPV16 and/or 18 Positivity was associated with a higher proportion of HSIL+ diagnoses than positivity for the 12 Other HPV types (56.5% vs. 33.9%; p=0.061). Statistics: Chi-square or Fisher exact test. Abbreviations: ASC-US, atypical squamous cells of undetermined significance; LSIL, low-grade squamous intraepithelial lesion; ASC-H, atypical squamous cells, cannot exclude HSIL; HSIL, high-grade squamous intraepithelial lesion; AGC, Atypical glandular cells; AIS, adenocarcinoma in situ.

Table 3 shows the performance of HPV testing and LBC, individually and in combination. Considering all 227 evaluated cases (including two invalid samples without HSIL, classified as FP), HPV testing alone demonstrated the highest sensitivity (94.3%), identifying nearly all HSIL+ cases (33/35), but with lower specificity (73.4%) and a higher number of FP results (49 cases). The PPV of 39.3% indicates that approximately 40 of every 100 positive HPV tests corresponded to HSIL+ diagnosis. HPV testing also showed a high NPV (98.6%), effectively minimizing the likelihood of HSIL+ after a negative result. LBC alone demonstrated the highest overall accuracy (84.1%), with a sensitivity of 80%, detecting fewer HSIL+ cases (28/35) than HPV testing, but with improved specificity to 84.9%. Compared with HPV testing, LBC yielded fewer FP results (27 vs. 49) and a 10% higher PPV (49.1%), meaning that approximately one in two positive LBC results correspond to HSIL+. The NPV of 95.9% was also highly reliable for excluding HSIL+.

**Table 3:** Performance of HPV testing and/or Liquid-Based Cytology (LBC) alone or in combination for HSIL+ detection in 227* women with negative colposcopy

Test	HSIL+ (N)	SENS % (n/N)	SPEC % (n/N)	PPV % (n/N)	NPV % (n/N)	ACCU %
HPV	33	94.3 (33/35)	73.4 (141/192)	39.3 (33/84)	98.6 (141/143)	76.6
LBC	28	80.0 (28/35)	84.9 163/192)	49.1 (28/57)	95.9 (163/169)	84.1
HPV "or" LBC	34	97.1 (34/35)	66.7 (128/192)	34.7 (34/98)	99.2 (128/129)	71.4
HPV "and" LBC	27	77.1 (27/35)	91.7 176/192)	62.8 (27/43)	95.7 (176/184)	89.4

* Two cases (0.9%), one for each test, had invalid or unsatisfactory samples, all without HSIL+ and counted as FP. Abbreviations: SENS, sensitivity; SPEC, specificity; PPV/NPV, positive/negative predictive value; ACCU, accuracy

When HPV testing and LBC were analyzed in combination, two scenarios were evaluated. In the first scenario, positivity was defined when either test was positive (*"OR"* rule). This approach detected 34 of 35 HSIL+ cases (sensitivity 97.1%) and achieved an NPV of 99.2%, with only one FN result. However, specificity dropped to 66.7% and PPV to 34.7%, indicating a higher likelihood of unnecessary additional procedures.

In the second scenario, positivity was defined only when both tests were positive (*"AND"* rule). This approach significantly improved specificity (91.7%), PPV (62.8%, the highest observed), and overall accuracy (89.4%), but reduced sensitivity to 77.1% (27/35 HSIL+ detected). The eight FN cases included clinically significant diagnoses: five CIN3 and one microinvasive carcinoma (HPV+/LBC–), one CIN3 (HPV–/LBC+), and one CIN3 (HPV–/LBC–).

## Discussion

This study addressed a well-recognized diagnostic challenge: the management of women screened with ASC-H or worse cytology results but negative colposcopy findings. In this group, 15.4% of HSIL+ cases were detected, including two superficially invasive carcinoma, reinforcing the need for additional investigation to identify potential occult lesions. In the literature, the prevalence of HSIL in negative colposcopies ranges from 10% to 40%.^(16-18)^

HPV testing or LBC, when used as additional tests, achieved a maximum HSIL+ detection rate of 97.1% (34/35). However, this increase in sensitivity was accompanied by reduced specificity and PPV, potentially increasing the number of unnecessary excisional procedures.

Considering a single additional test after negative colposcopy, HPV testing showed higher sensitivity (94.3%) and NPV (98.6%) than LBC, confirming its excellent utility in excluding disease when negative. However, HPV specificity (73.4%) and PPV (39.3%) were lower, as expected, reflecting a greater number of FP, and can potentially leading a higher number of unnecessary procedures.^(19-21)^ Importantly, in presence of HPV16 and/or 18 genotypes, specificity improved to 94.8% and PPV increased to 56.5%, underscoring the genotyping value in guiding more precise clinical management.^(22,23)^

By contrast, LBC alone, essentially a repetition of the screening cytology, showed a more balanced performance, with sensitivity of 80%, specificity of 84.9%, overall accuracy of 84.1%, and PPV of 49.1%, which may aid clinical decision making in cases of negative colposcopy.^(24)^ These findings are consistent with previous studies showing that, although cytology is less sensitive, its higher specificity and PPV may be advantageous in this context.

Given the results so far, it stands to reason that using both tests in combination can improve overall performance and optimize the balance between FP and FN. This approach is similar to the screening cotesting strategy but applied here after a negative colposcopy. We tested the combination in two scenarios: when either test was positive (HPV "OR" LBC), and when both tests were positive (HPV "AND" LBC).

The strategy with HPV "OR" LBC positivity, sensitivity increased to 97.1% with nearly elimination of FN (NPV 99.2%). However, specificity decreased markedly (66.7%) and PPV dropped to 34.7%. This pattern suggests that applying a complementary positivity criterion (considered positive if either test was positive) enhances diagnostic safety when one test is negative (decreasing FN) but increases clinical workload due to a higher number of positive results in women without disease, potentially leading to overtreatment.

The restrictive combination strategy considered a case positive only when both HPV "AND" LBC were positive, yielded the best performance for confirming HSIL+, with specificity of 91.7%, PPV of 62.8%, and the highest overall accuracy (89.4%). However, this improvement came at the cost of lower sensitivity (77.1%), increasing the risk of missed HSIL+ cases. Importantly, FN results included high-grade lesions and even microinvasive carcinoma, highlighting that this strategy cannot guarantee complete diagnostic safety and should always be interpreted in the contest of careful clinical follow-up in this setting. Despite this limitation, when both tests are positive, performing an excision of the transformation zone (EZT) may be strongly considered and justified in the appropriate clinical context.

Overall, regarding diagnostic safety, the results reinforce the role of HPV testing as the best tool for ruling out HSIL+ (NPV 98.6%), while adding LBC slightly improves the NPV (99.2%).^(25,26)^ These findings suggest that they can guide the selection of women who may safely defer excisional procedures under careful follow-up. Conversely, for greater accuracy in detecting HSIL+, a positive LBC alone increased PPV from 39.3% (HPV test), to 49.1%, and when both tests were positive ("HPV-LBC"), the PPV reached 62.8%, potentially reducing unnecessary interventions.

The choice of diagnostic strategy should balance safety, minimizing FN, and efficiency, reducing FP and unnecessary procedures, and incorporating additional tests targeting the endocervix may help and optimize this balance.

This study has several strengths, including its controlled pattern and multicenter design, standardized test application, use of EZT and/or rigorous follow-up as the reference standard, a low loss of follow-up rate, and alignment with the routine clinical practice of the Brazilian healthcare system. Limitations include the time interval between screening cytology and study inclusion (up to 12 months, though ≤ 120 days for 80% of cases), although, an interval of up to 120 days reflects the current dynamics with Brazilian healthcare system, where delays between screening results and colposcopy evaluation may occur in routine practice. Nevertheless, regression or progression of lesions during this period cannot be completely excluded and should be considered when interpreting cytology-histology correlations and diagnostic performance estimates. Not all participants underwent an excisional procedure for histological confirmation because such an approach would have been ethically unjustified. Therefore, negative cases were confirmed by 18-month follow-up, which may underestimate slowly progressive lesions. The study findings should be interpreted within the context of real-world clinical practice in referral service and cannot be extrapolated to cases with a positive colposcopy.

In recent years, cervical cancer prevention strategies have increasingly incorporated risk-based management approaches, such as those proposed by the ASCCP guidelines.^(6)^ In this context, clinical decisions are guided by the estimated risk of CIN3 or worse rather than isolated test results. Although the present study focused on traditional measures of diagnostic performance, our findings are aligned with this paradigm. The high NPV observed for the HPV test, particularly when combined with cytology, suggests that these tests may help identify women with a sufficiently low risk of HSIL+ after negative colposcopy, potentially supporting more conservative follow-up strategies while maintaining patient safety.

Besides the increasing adoption of risk-based management strategies, there is an ongoing modernization of cervical cancer screening, with primary HPV testing supported by reflex LBC, and our findings are useful for both, providing clinically relevant evidence for incorporating additional testing after negative colposcopy in women with high-grade cytological abnormalities. These data may help refine risk stratification and support more individualized clinical decision-making.

## Conclusion

Even after negative colposcopy, women with ASC-H, HSIL, or AGC screening cytology remain at substantial risk for underlying high-grade cervical lesions. In this scenario, HPV DNA testing showed high diagnostic value for ruling out HSIL+, supported by its high negative predictive value. The complementary use of HPV testing and LBC improved diagnostic performance, reducing false-negative results and increasing diagnostic accuracy when both tests were positive. This combined approach may help guide clinical management after negative colposcopy by improving risk stratification while helping to avoid unnecessary excisional procedures. These findings support the complementary use of HPV testing and cytology in the evaluation of women with high-risk cytology and negative colposcopy, particularly in settings transitioning from cytology-based to primary HPV-based cervical screening.

## Data Availability

The research data are described in the article presented.
